# Effects of Dietary *Wedelia chinensis* (Osbeck) Merr. Extract on Growth Performance, Feed Utilization, Antioxidant Status, and Innate Immunity in Nile Tilapia (*Oreochromis niloticus*)

**DOI:** 10.3390/ani16131986

**Published:** 2026-06-27

**Authors:** Chinh Xuan Le, Tran Thi Nang Thu, Le Thi Hoang Hang, Ha Thi Thanh Nguyen, Manh Duc Vu, Thi Mai Nguyen

**Affiliations:** 1 Faculty of Fisheries, Vietnam National University of Agriculture, Hanoi 131000, Vietnam; trannangthu@vnua.edu.vn (T.T.N.T.); lethihoanghang@gmail.com (L.T.H.H.); manhducvu.1994@gmail.com (M.D.V.); ntmai.ntts@vnua.edu.vn (T.M.N.); 2 Science and Technology Office, Vietnam National University of Agriculture, Hanoi 131000, Vietnam; 3 Department of Veterinary Pharmacology, Faculty of Veterinary Medicine, Vietnam National University of Agriculture, Hanoi 131000, Vietnam; nguyenhavet@vnua.edu.vn

**Keywords:** *Wedelia chinensis* extract, Nile tilapia, plant-derived additive, growth performance, antioxidant status, innate immunity, feed utilization, gene expression

## Abstract

Plant-derived additives are increasingly explored as functional aquafeed ingredients for supporting fish performance and physiological condition. This study evaluated the effects of dietary *Wedelia chinensis* extract (WCE) on growth performance, feed utilization, antioxidant status, innate immune-associated biomarkers, and immune- and antioxidant-related gene expression in Nile tilapia (*Oreochromis niloticus*). Juvenile fish with an initial body weight of 14.31 ± 0.03 g were fed diets supplemented with WCE at 0, 5, 10, 15, or 20 g kg^−1^ diet for eight weeks. Growth indices, somatic indices, serum and hepatic antioxidant biomarkers, mucosal and systemic innate immune-associated parameters, and liver and intestine gene expression were assessed. Dietary WCE improved final weight, weight gain rate, specific growth rate, and feed conversion ratio compared with the control, with WCE10 showing the most consistent growth-related response. Somatic indices were not significantly affected. WCE supplementation increased antioxidant enzyme activities and reduced malondialdehyde levels in serum and liver, particularly at moderate inclusion levels. It also increased lysozyme and peroxidase activities, alternative complement activity, phagocytic index, and respiratory burst activity, and modulated selected immune- and antioxidant-related genes in the liver and intestine. Taken together, dietary WCE, particularly at 10 g kg^−1^ diet, improved growth and feed utilization while supporting antioxidant status and basal innate immune-associated biomarkers during the eight-week feeding trial, without detectable changes in somatic indices. These findings provide a basis for further evaluating WCE as a plant-derived functional additive for Nile tilapia aquafeeds under pathogen-challenge and practical farming conditions.

## Simple Summary

Nile tilapia is an important aquaculture species, but intensive culture conditions may impair growth efficiency, oxidative balance, and basal defense responses. Plant-derived feed additives are therefore being explored as nutritional strategies to support fish performance and physiological condition. This study evaluated the effects of dietary *Wedelia chinensis* extract in juvenile Nile tilapia with an initial body weight of approximately 14.3 g. Fish were fed diets containing 0, 5, 10, 15, or 20 g kg^−1^ of the extract for eight weeks. Dietary *W. chinensis* extract improved growth performance and feed utilization compared with the control diet and was accompanied by increased antioxidant enzyme activities, reduced lipid peroxidation, and favorable changes in mucosal, humoral, and cellular innate immune-associated biomarkers. The most consistent overall response was observed in fish fed 10 g kg^−1^ diet, whereas higher inclusion levels did not result in further overall improvement. These findings suggest that *W. chinensis* extract may be considered a plant-derived functional additive for Nile tilapia diets, although further validation under pathogen-challenge and practical farming conditions is still needed.

## 1. Introduction

Tilapia has become a globally important aquaculture commodity, with farming reported in more than 100 countries and global production estimated at approximately 7.4 million tons in 2024 [1]. Nile tilapia (*Oreochromis niloticus*) is the most widely farmed tilapia species because of its rapid growth, efficient feed utilization, broad environmental tolerance, adaptability to different farming systems, and strong market demand [2]. However, the continued expansion and intensification of Nile tilapia production expose fish to husbandry-related stressors, including high stocking density, fluctuating water quality, oxidative imbalance, and disease pressure [3,4]. These factors can impair growth performance, feed utilization, physiological stability, and basal immune function, thereby reducing production efficiency. Although antibiotics and chemotherapeutic agents have historically been used to control bacterial diseases in aquaculture, their extensive use has raised concerns regarding antimicrobial resistance, drug residues, environmental contamination, and disruption of host-associated microbiota [5,6]. Therefore, sustainable feed-based strategies that support fish performance and physiological condition while reducing reliance on conventional antimicrobial interventions are increasingly needed.

Plant-derived additives have attracted considerable attention as functional aquafeed ingredients because they contain diverse bioactive compounds that may support growth, antioxidant defense, immune-associated responses, and intestinal function in fish [7,8]. Compared with raw plant powders, extracts may provide a more concentrated source of soluble phytochemical fractions, although their biological efficacy depends on plant species, extraction procedure, inclusion level, feeding duration, fish species, and culture conditions [9]. *Wedelia chinensis* (Osbeck) Merr. is a medicinal plant belonging to the family Asteraceae and has been widely used in traditional medicine in several Asian countries. Previous phytochemical studies have reported that *W. chinensis* contains phenolic and flavonoid compounds together with other secondary metabolites, which are generally associated with antioxidant, anti-inflammatory, antimicrobial, hepatoprotective, and wound-healing properties [10,11,12]. These characteristics suggest that *W. chinensis* may have potential as a phytogenic feed additive; however, its biological effects should be evaluated in a species-specific and dose-dependent manner [13,14]. In aquaculture, information on *W. chinensis* remains limited and is mainly derived from shrimp studies. Dietary *W. chinensis* extract has been reported to improve growth performance, feed utilization, antioxidant enzyme activity, innate immune responses, and post-challenge outcomes in whiteleg shrimp (*Penaeus vannamei*) [15,16]. Nevertheless, evidence in finfish is still scarce, and little is known about how dietary *W. chinensis* extract affects growth performance, oxidative status, innate immune-associated biomarkers, and related gene expression in Nile tilapia. This knowledge gap limits the practical evaluation of *W. chinensis* as a functional additive for tilapia feeds.

Therefore, the present study evaluated the effects of dietary *W. chinensis* extract (WCE) on growth performance, feed utilization, somatic indices, antioxidant and oxidative stress biomarkers, innate immune-associated responses, and the relative expression of selected immune- and antioxidant-related genes in Nile tilapia. The study aimed to provide experimental evidence for the potential use of WCE as a plant-derived functional additive in Nile tilapia aquafeeds.

## 2. Materials and Methods

### 2.1. Preparation of W. chinensis Extract

Fresh whole-plant material of *W. chinensis* was collected from Vietnam National University of Agriculture, Hanoi, Vietnam. The plant material was cleaned, washed thoroughly with tap water, rinsed with distilled water, and dried in a hot-air oven at 40 °C until a constant weight was obtained. The dried material was ground into powder and passed through a 100-mesh sieve before extraction.

WCE was prepared by maceration following Nguyen et al. (2024), with minor modifications [17]. Briefly, the powdered material was extracted with 96% ethanol at a solid-to-solvent ratio of 1:30 (*w*/*v*) in the dark at room temperature for 24 h, with brief stirring before and after extraction. The extraction was performed in three independent batches, and the obtained crude extracts were combined to prepare a homogeneous WCE batch for diet preparation. The mixture was centrifuged at 3500 rpm for 10 min at room temperature, and the supernatant was filtered through qualitative filter paper. The filtrate was then concentrated under reduced pressure at ≤40 °C to remove ethanol and obtain the crude WCE. The extract was stored at −20 °C until use in experimental diet preparation. The crude extract used in the feeding trial was characterized based on extraction yield, total phenolic content, total flavonoid content, and DPPH radical-scavenging activity. These parameters were used as basic quality-control indicators for the crude WCE batch and are presented in Table 1. No further purification or fractionation was applied, as the present study aimed to evaluate the biological effects of crude WCE as a practical plant-derived feed additive.

### 2.2. Experimental Diets

Experimental diets were formulated based on a Nile tilapia diet previously used in feeding trials with plant-derived additives with minor modifications [18]. Because no prior dietary dose–response information for *W. chinensis* extract in Nile tilapia was available, the 0–20 g kg^−1^ diet range was selected as an exploratory range based on previous tilapia studies of plant-derived additives and practical feed formulation considerations. This range corresponded to 0–2% of the diet, allowed WCE to replace wheat flour, and used 5 g kg^−1^ increments to evaluate potential dose-related responses. Five experimental diets were prepared by incorporating *W. chinensis* extract (WCE) into the basal formulation at 0, 5, 10, 15, and 20 g kg^−1^ diet and were designated as WCE0, WCE5, WCE10, WCE15, and WCE20, respectively. The inclusion level of wheat flour was adjusted to maintain the total formulation at 1000 g kg^−1^ diet. The formulation and analyzed proximate composition of the diets are presented in Table 2. All dry ingredients were thoroughly mixed before the addition of WCE, soybean oil, and water to form a homogeneous dough. To ensure uniform distribution, WCE was first premixed with a small portion of the basal ingredients before being incorporated into the complete mixture. After WCE incorporation, the diets were pelleted without further heat treatment, air-dried at room temperature, sealed in plastic bags, and stored at 4 °C until use.

### 2.3. Experimental Fish and Feeding Trial

Apparently healthy Nile tilapia fingerlings were obtained from a local hatchery and acclimated to experimental conditions for two weeks, during which they were fed a commercial diet twice daily. After acclimation, fish with an average initial body weight of 14.31 ± 0.03 g were randomly distributed into 15 fiberglass tanks with a capacity of 300 L, at a density of 20 fish per tank. The experiment was arranged in a completely randomized design with five dietary treatments and three replicate tanks per treatment.

The feeding trial lasted 8 weeks. Fish were hand-fed twice daily at 4% of body weight per day, with the daily ration divided equally between the two meals. Feed was offered gradually while feeding behavior was monitored to minimize waste. Visible uneaten pellets were removed by siphoning after feeding to maintain water quality. Fish in each tank were bulk-weighed every two weeks, and the feeding ration was adjusted accordingly. Because uneaten feed was not quantitatively recovered, feed intake for FCR calculation was based on the amount of dry feed offered to each tank. Water quality was monitored throughout the trial. Temperature, pH, and dissolved oxygen were measured twice daily using a multiparameter meter (HI98196, Hanna Instruments, Cluj-Napoca, Romania), whereas total ammonia nitrogen was determined using a portable ammonia photometer (HI96733, Hanna Instruments, Cluj-Napoca, Romania). During the experiment, water temperature, pH, dissolved oxygen, and total ammonia nitrogen were maintained at 28.50 ± 0.50 °C, 7.82 ± 0.32, 5.07 ± 0.04 mg L^−1^, and 0.12 ± 0.02 mg L^−1^, respectively. Approximately 20% of the tank water was exchanged daily to maintain water quality. All procedures involving fish were conducted in accordance with institutional guidelines for the care and use of experimental animals. The feeding trial was conducted under the approved Vietnam National University of Agriculture research project “Effects of dietary herbal extracts on growth performance, immune responses, and gut microbiota of Nile tilapia (*Oreochromis niloticus*)”, code T2024-13-22TĐ, which was reviewed by the institutional scientific committee established under Decision No. 352/QĐ-HVN dated 26 March 2024.

### 2.4. Growth Performance and Somatic Indices

Individual body weight was recorded at the beginning of the experiment and at weeks 4 and 8 of the feeding trial. Growth performance indices were calculated as follows: weight gain rate (WGR, %) = 100 × (FW − IW)/IW; specific growth rate (SGR, % day^−1^) = 100 × [ln(FW) − ln(IW)]/t, where t is the feeding duration in days; survival rate (SR, %) = 100 × final fish number/initial fish number; and feed conversion ratio (FCR) = dry feed intake/wet weight gain.

At the end of the feeding trial, fish were dissected, and the liver, viscera, and intestine were weighed to determine somatic indices. The hepatosomatic index (HSI, %) was calculated as 100 × liver weight/final body weight; the viscerosomatic index (VSI, %) as 100 × viscera weight/final body weight; and the intestinosomatic index (ISI, %) as 100 × intestine weight/final body weight.

### 2.5. Sampling Procedures and Sample Preparation

At weeks 4 and 8, three fish from each tank were randomly collected after a 24-h fasting period and anesthetized with clove oil (30 mg L^−1^). Skin mucus and serum were sampled following Van Doan et al. (2025), whereas leukocytes were isolated according to Le Xuan et al. (2024), with minor modifications [18,19]. Fish sampled from the same tank were treated as biological subsamples and not as independent experimental units. For assays performed in technical triplicate, the mean of the technical replicates was used as the individual fish value. The values obtained from the three fish sampled within each tank were subsequently averaged to generate one tank mean for each parameter.

For skin mucus collection, each fish was individually transferred to a sterile plastic bag containing 10 mL of 50 mM NaCl. The body surface was gently massaged for 1 min to release mucus into the saline solution. The resulting mucus suspension was immediately collected, maintained on ice during processing, and stored at −80 °C until analysis. For serum preparation, blood was withdrawn from the caudal vein without anticoagulant and allowed to clot for 1 h at room temperature followed by 4 h at 4 °C. The clotted blood was then centrifuged at 10,000× *g* for 15 min at 4 °C, and the separated serum was collected and stored at −80 °C.

For leukocyte preparation, a separate blood sample was collected using sodium heparin-treated syringes. The heparinized blood was diluted twofold with RPMI-1640 medium and carefully layered onto Histopaque. After centrifugation at 400× *g* for 30 min at 25 °C, the leukocyte-enriched layer was recovered, washed twice with phosphate-buffered saline at 250× *g* for 10 min, and finally resuspended in RPMI-1640 medium for subsequent phagocytic activity and respiratory burst assays.

Following mucus and blood sampling, fish were dissected for somatic index determination and tissue collection. The liver, visceral mass, and intestine were excised and weighed to calculate the hepatosomatic, viscerosomatic, and intestinosomatic indices. For antioxidant assays, liver tissue was rinsed with ice-cold physiological saline, homogenized at a ratio of 1:9 (*w*/*v*), and centrifuged at 2500× *g* for 10 min at 4 °C. The supernatant was collected for biochemical analyses. Additional liver and intestine samples were placed in TRIzol reagent and stored at −80 °C until RNA extraction.

### 2.6. Antioxidant Assays

Antioxidant enzyme activities and lipid peroxidation levels were determined in serum and liver homogenates using commercial assay kits according to the manufacturers’ instructions. Superoxide dismutase (*SOD*), catalase (*CAT*), and malondialdehyde (MDA) were measured using assay kits from Nanjing Jiancheng Bioengineering Institute (Nanjing, China). *SOD* activity was determined using the hydroxylamine method, in which the inhibition of nitrite formation was measured spectrophotometrically at 550 nm. *CAT* activity was measured using the visible-light ammonium molybdate method, based on the decomposition of hydrogen peroxide, with absorbance recorded at 405 nm. MDA concentration, as an indicator of lipid peroxidation, was determined using the thiobarbituric acid reactive substances method, and absorbance was measured at 532 nm.

Glutathione peroxidase (*GPx*) activity in liver homogenates was determined using a commercial assay kit (E-BC-K809-M, Elabscience, Houston, TX, USA) following the manufacturer’s protocol. The assay was based on the enzymatic reduction of peroxide, monitored by the decrease in absorbance at 340 nm. Hepatic enzyme activities were normalized to total protein concentration in the liver homogenates. Results were expressed as U mL^−1^ serum for serum *SOD* and *CAT* activities, U mg^−1^ protein for hepatic *SOD* and *CAT* activities, U g^−1^ protein for hepatic *GPx* activity, and nmol mL^−1^ serum or nmol mg^−1^ protein for MDA concentration, as appropriate.

### 2.7. Innate Immune Assays

Lysozyme activity in serum and skin mucus was determined using the turbidimetric method of Parry Jr et al. (1969), with minor modifications as described by Outama et al. (2022) [20,21]. For each reaction, 100 μL of skin mucus or 25 μL of serum was added to a *Micrococcus lysodeikticus* suspension prepared in citrate–phosphate buffer. The reduction in turbidity was monitored at 540 nm every 30 s for 5 min at 25 °C using a microplate reader. Lysozyme activity was calculated using a hen egg-white lysozyme standard curve and expressed as μg mL^−1^.

Peroxidase activity in serum and skin mucus was measured according to Quade and Roth (1997), with minor modifications by Le Xuan et al. (2024) [18,22]. Briefly, each sample was combined with Ca^2+^/Mg^2+^-free Hanks’ balanced salt solution and freshly prepared TMB–H_2_O_2_ substrate solution. The enzymatic reaction was terminated with 2 M H_2_SO_4_, and absorbance was read at 450 nm. After blank correction, peroxidase activity was expressed as U mL^−1^.

Alternative complement pathway activity (ACH50) was determined using the modified hemolytic method of Yanno (1992), with minor modifications by Le Xuan et al. (2024) [18,23]. Rabbit red blood cells were prepared in EGTA–Mg–GVB buffer and incubated with serially diluted serum samples at 20 °C for 1.5 h. The mixture was then centrifuged, and the absorbance of the supernatant was measured at 414 nm. ACH50 activity was expressed as U mL^−1^.

Cellular innate immune responses were assessed using leukocyte suspensions prepared from peripheral blood. Phagocytic activity was determined according to Yoshida and Kitao (1991), with modifications described by Van Doan et al. (2018) [24,25]. Leukocytes were allowed to adhere to coverslips and incubated with fluorescent latex beads. After washing, fixation, staining, and mounting, adherent cells were examined under a microscope, and phagocytic activity was expressed as the phagocytic index.

Respiratory burst activity was assessed using the nitroblue tetrazolium reduction assay according to A. Tahir and C. Secombes (1996), with minor modifications as described by Van Doan et al. (2018) [25,26]. Leukocyte suspensions were incubated with NBT solution, and the resulting formazan was dissolved using dimethyl sulfoxide and KOH. Absorbance was recorded at 655 nm, and respiratory burst activity was expressed as OD_655_.

### 2.8. Gene Expression Analysis

The relative expression of selected immune-related genes, including *IL-1β*, *IL-8*, and *LBP*, and antioxidant-related genes, including *GSTα*, *GPX*, and *GSR*, was analyzed in liver and intestine samples collected at the end of the 8-week feeding trial. Approximately 25–50 mg of each tissue sample was homogenized in TRIzol reagent (Life Technologies, Carlsbad, CA, USA) using sterile pellet pestles. After phase separation with chloroform, the aqueous phase containing RNA was collected and further purified using the PureLink™ RNA Mini Kit (Invitrogen, Austin, TX, USA), according to the manufacturer’s instructions.

RNA concentration and purity were assessed using a NanoDrop™ spectrophotometer (Thermo Scientific, Waltham, MA, USA) based on the A260/A280 and A260/A230 absorbance ratios. RNA samples used for cDNA synthesis showed A260/A280 and A260/A230 ratios of 1.88–2.07 and 1.79–2.20, respectively (Supplementary Table S2). First-strand cDNA was synthesized from 500 ng of total RNA using the iScript™ cDNA Synthesis Kit (Bio-Rad, Hercules, CA, USA). Quantitative real-time PCR was performed using a CFX Connect™ Real-Time PCR System (Bio-Rad, Hercules, CA, USA) in a final reaction volume of 20 μL containing cDNA template, gene-specific primers, 2× Universal SYBR Green Supermix, and nuclease-free water. All reactions were run in technical triplicate. The amplification conditions followed [27] with minor modifications. Five-point cDNA dilution curves yielded primer efficiencies of 95.2–103.8% (R^2^ = 0.9996–1.0000), which were comparable between the target genes and the *18S rRNA* reference assay (Supplementary Table S1). Amplification specificity was assessed by melting-curve analysis, which showed a predominant gene-specific peak for each primer pair (Supplementary Figure S4). Relative gene expression was calculated using the 2^−ΔΔCt^ method (Livak and Schmittgen, 2001 [28]), with 18S rRNA used as the reference gene [28]. Its stability was confirmed at the tank level, as 18S rRNA Ct values did not differ among dietary treatments in either liver or intestine (*p* > 0.05; Supplementary Table S2). Primer sequences are presented in Table 3.

### 2.9. Statistical Analysis

All data were analyzed using Minitab Statistical Software version 21.4. The tank was considered the experimental unit for all statistical analyses. For fish-based measurements, where applicable, technical replicates were first averaged to obtain one value for each individual fish, and the values from the three fish sampled within the same tank were then averaged to obtain one tank mean. Only tank means were included in the statistical analysis (*n* = 3 tanks per dietary treatment); technical replicates were not treated as independent observations. Data were checked for normality using the Anderson–Darling test and for homogeneity of variance using Levene’s test. Differences among dietary treatments were analyzed by one-way analysis of variance (ANOVA), followed by Tukey’s multiple comparison test when significant differences were detected. Statistical significance was accepted at *p* < 0.05. Polynomial regression analysis was used to evaluate dose–response relationships between dietary WCE inclusion levels and selected response variables. Additional polynomial regression plots for antioxidant and innate immune-associated parameters are provided in Supplementary Figures S1–S3.

## 3. Results

### 3.1. Growth Performance

The effects of dietary WCE supplementation on growth performance, feed utilization, and survival of Nile tilapia are presented in Table 4. Initial weight did not differ among treatments, indicating comparable fish distribution at the start of the trial. Dietary WCE significantly improved final weight, weight gain rate, specific growth rate, and feed conversion ratio at both week 4 and week 8 (*p* < 0.05). At week 4, fish fed WCE5, WCE10, and WCE15 showed significantly higher final weight, weight gain rate, and specific growth rate than the control, whereas WCE20 generally showed an intermediate response. Feed conversion ratio was significantly lower in all WCE-supplemented groups than in WCE0 (*p* < 0.05). After 8 weeks, all WCE-supplemented groups exhibited significantly higher final weight, weight gain rate, and specific growth rate, together with a lower feed conversion ratio, compared with the control group (*p* < 0.05). Among the supplemented groups, WCE10 showed the highest numerical values for final weight, weight gain rate, and specific growth rate, as well as the lowest feed conversion ratio. Survival remained high throughout the feeding trial and was not significantly affected by dietary treatment (*p* > 0.05). Overall, dietary WCE improved growth performance and feed utilization, with the most consistent response observed at 10 g kg^−1^ diet.

**Figure 1:**
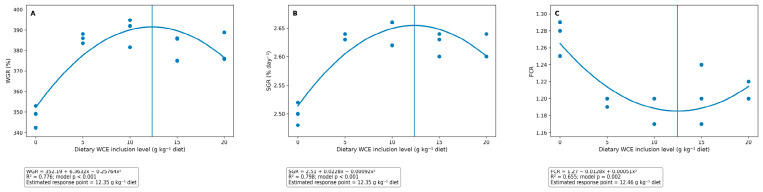
Quadratic regression relationships between dietary *Wedelia chinensis* extract (WCE) inclusion level and weight gain rate (WGR; (**A**)), specific growth rate (SGR; (**B**)), and feed conversion ratio (FCR; (**C**)) in Nile tilapia after the 8-week feeding trial. Each point represents one tank (*n* = 3 tanks per dietary treatment). Solid curves represent fitted quadratic regression models, and vertical lines indicate the estimated response points. Regression equations, coefficients of determination (R^2^), and overall model *p* values are presented in the corresponding panels.

Polynomial regression analysis further showed significant linear and quadratic responses of WGR, SGR, and FCR to dietary WCE inclusion (Figure 1). At week 4, both linear and quadratic effects were significant for WGR, SGR, and FCR (*p* < 0.01). At week 8, WGR and SGR increased with WCE inclusion up to an intermediate level and then declined slightly at higher levels, whereas FCR showed the opposite curvilinear pattern. The estimated response points were 12.35 g kg^−1^ diet for WGR, 12.35 g kg^−1^ diet for SGR, and 12.46 g kg^−1^ diet for FCR. These results indicate that growth and feed utilization responses to WCE were non-linear, with the strongest responses occurring around 12–13 g kg^−1^ diet.

### 3.2. Somatic Indices

Somatic indices of Nile tilapia after 8 weeks of feeding are presented in Table 5. Dietary WCE supplementation did not significantly affect hepatosomatic index, viscerosomatic index, or intestinosomatic index among treatments (*p* > 0.05). Although HSI and VSI showed slight numerical increases in fish fed WCE10, these differences were not statistically significant. Overall, WCE inclusion up to 20 g kg^−1^ diet did not adversely affect major somatic indices of Nile tilapia.

### 3.3. Antioxidant Activities

The effects of dietary WCE supplementation on serum and hepatic antioxidant status are shown in Table 6. Dietary WCE significantly affected antioxidant enzyme activities and MDA levels in both serum and liver (*p* < 0.05). In serum, *CAT* and *SOD* activities were highest in the WCE10 group and were significantly higher than those in the control group (*p* < 0.05), whereas the other WCE-supplemented groups generally showed intermediate values. Serum MDA levels were reduced in WCE-supplemented fish, with significant decreases observed in the WCE10 and WCE15 groups compared with the control group (*p* < 0.05). In the liver, *CAT* and *SOD* activities were also highest in fish fed the WCE10 diet and were significantly higher than those in the control group (*p* < 0.05). Hepatic *GPx* activity was significantly increased in the WCE10, WCE15, and WCE20 groups compared with the control group (*p* < 0.05). Conversely, hepatic MDA levels were lowest in WCE10 and significantly lower than those in the control group (*p* < 0.05). Overall, dietary WCE improved antioxidant status and reduced lipid peroxidation in Nile tilapia, with the most consistent response observed in the WCE10 group. Additional polynomial regression analyses for antioxidant parameters are presented in Supplementary Figure S1. The fitted curves generally showed curvilinear responses to dietary WCE supplementation, with estimated response points distributed within approximately 8–15 g kg^−1^ diet. These trends were consistent with the treatment-based results, in which the most favorable antioxidant responses were mainly observed at moderate WCE inclusion levels rather than at the highest dietary level.

### 3.4. Innate Immune-Related Parameters

The effects of dietary WCE on innate immune-associated parameters are presented in Table 7. After 4 weeks, WCE supplementation significantly affected mucosal and serum immune enzyme activities, ACH50, and respiratory burst activity (*p* < 0.05), whereas the phagocytic index was not significantly changed (*p* > 0.05). The WCE10 group generally showed the most pronounced responses, with higher lysozyme and peroxidase activities, ACH50, and respiratory burst activity than the control group (*p* < 0.05). After 8 weeks, all measured immune-associated parameters were significantly affected by dietary treatment (*p* < 0.05), and WCE10 remained among the best-performing groups across mucosal, humoral, and cellular parameters. Overall, WCE supplementation improved innate immune-associated biomarkers, with the most consistent response observed at 10 g kg^−1^ diet. Polynomial regression analyses further showed curvilinear responses to dietary WCE supplementation (Supplementary Figures S2 and S3). The estimated response points were generally within 10–15 g kg^−1^ diet at week 4 and 10–14 g kg^−1^ diet at week 8, supporting a moderate inclusion range for immune-associated responses.

### 3.5. Relative Immune- and Antioxidant-Related Gene Expression

**Figure 2:**
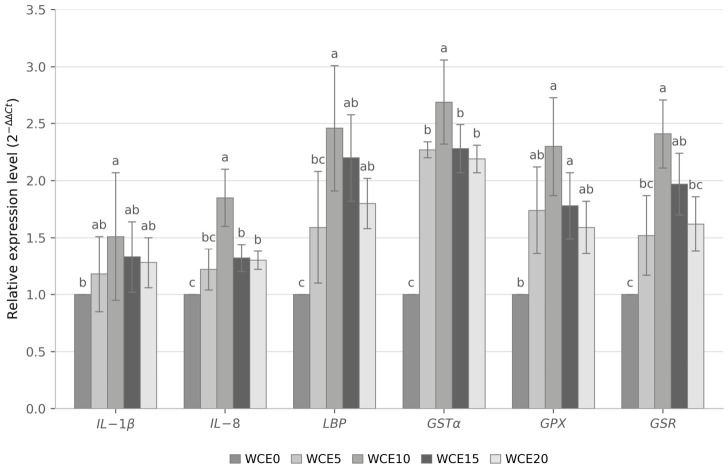
Relative expression levels of selected immune- and antioxidant-related genes in the liver of Nile tilapia fed diets supplemented with different levels of WCE after 8 weeks of feeding. Values are presented as mean ± SE (*n* = 3). Different letters above bars indicate significant differences among treatments within the same gene (*p* < 0.05). Relative gene expression was normalized to 18S rRNA and calibrated against the WCE0 group. WCE, *W. chinensis* extract; *IL-1β*, interleukin-1 beta; *IL-8*, interleukin-8; *LBP*, lipopolysaccharide-binding protein; *GSTα*, glutathione S-transferase alpha; *GPX*, glutathione peroxidase; *GSR*, glutathione reductase.

**Figure 3:**
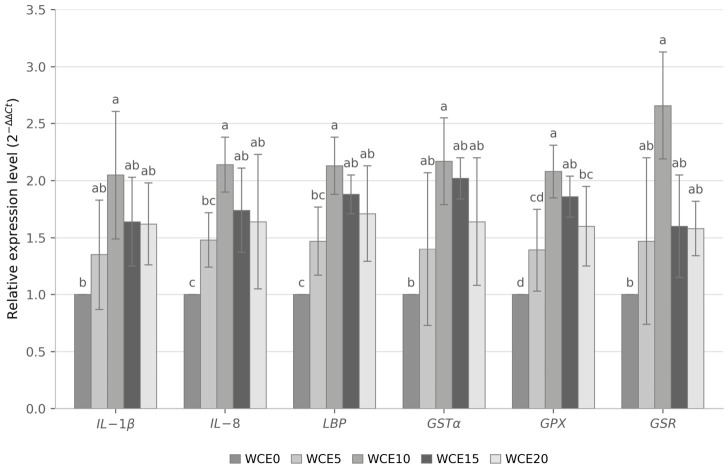
Relative expression levels of selected immune- and antioxidant-related genes in the intestine of Nile tilapia fed diets supplemented with different levels of WCE after 8 weeks of feeding. Values are presented as mean ± SE (*n* = 3). Different letters above bars indicate significant differences among treatments within the same gene (*p* < 0.05). Relative gene expression was normalized to 18S rRNA and calibrated against the WCE0 group. WCE, *W. chinensis* extract; *IL-1β*, interleukin-1 beta; *IL-8*, interleukin-8; *LBP*, lipopolysaccharide-binding protein; *GSTα*, glutathione S-transferase alpha; *GPX*, glutathione peroxidase; *GSR*, glutathione reductase.

The relative expression levels of selected immune- and antioxidant-related genes in the liver and intestine are shown in Figure 2 and Figure 3, respectively. In the liver, dietary WCE significantly affected the expression of immune-related genes, including *IL-1β*, *IL-8*, and *LBP*, and antioxidant-related genes, including *GSTα*, *GPX*, and *GSR* (*p* < 0.05). For most target genes, the highest or among the highest expression levels were observed in fish fed the WCE10 diet. At higher inclusion levels, gene expression generally tended to decline, although several genes remained higher than those in the control group.

In the intestine, dietary WCE significantly affected the relative expression of *IL-8*, *LBP*, *GSTα*, *GPX*, and *GSR* (*p* < 0.05), with WCE10 generally showing the most pronounced response. In contrast, intestinal *IL-1β* expression showed only a numerical increase in WCE-supplemented groups and did not differ significantly among treatments (*p* > 0.05). Overall, dietary WCE influenced the transcriptional responses of selected immune- and antioxidant-related genes in a tissue-dependent manner, with the most consistent responses observed at 10 g kg^−1^ diet.

## 4. Discussion

The dietary application of *W. chinensis* extract in finfish remains largely unexplored, despite its reported phytochemical characteristics and biological activities. In this context, the present study provides new evidence that WCE may serve as a plant-derived additive for Nile tilapia by improving growth performance and feed utilization, supporting antioxidant status and innate immune-associated biomarkers, and modulating selected immune- and antioxidant-related genes without significantly affecting somatic indices. Notably, these responses were not strictly dose-dependent. Among the tested diets, WCE10 produced the most consistent overall responses, while dose–response analysis estimated the response range for growth and feed utilization at approximately 12–13 g kg^−1^ diet. This pattern suggests that moderate WCE supplementation may provide greater benefits than either no supplementation or higher inclusion levels.

The higher final body weight, WGR, and SGR, together with the lower FCR, indicate that dietary WCE improved growth performance and feed utilization in Nile tilapia. Similar growth-promoting effects have been reported for other phytogenic additives in this species, including Assam tea extract, aquatic fern, *Annona squamosa* leaf extract and some fermented herbal extract [29,30,31,32]. The improved growth and feed utilization may reflect the contribution of bioactive fractions present in WCE to physiological conditions favorable for nutrient utilization and growth; however, because digestive enzyme activity, nutrient digestibility, gut microbiota, and intestinal morphology were not examined, these pathways should be considered plausible explanations rather than direct findings of the present study. The unchanged HSI, VSI, and ISI further suggest that the growth improvement was not accompanied by marked changes in hepatic, visceral, or intestinal relative weight. This may indicate that the improved growth and feed utilization occurred without excessive visceral or hepatic enlargement, suggesting relatively stable gross nutrient allocation and physiological condition. Importantly, the response to WCE was not strictly linear. WCE10 produced the most consistent growth response among the tested diets. Although quadratic regression estimated the response points for WGR, SGR, and FCR at approximately 12.3–12.5 g kg^−1^ diet, the overall results suggest that the beneficial effects of WCE on growth and feed utilization were most evident within a moderate inclusion range rather than at the highest tested levels.

Dietary WCE improved antioxidant status in both serum and liver, as shown by increased *SOD*, *CAT*, and *GPx* activities and reduced MDA levels. Similar responses have been reported in Nile tilapia fed *Annona squamosa* leaf extract and *Ocimum basilicum* extracts, which improved redox status, antioxidant balance, and related physiological responses [32,33]. In addition, dietary *Phyllanthus emblica* has been shown to upregulate antioxidant-related genes, including *SOD* and *GPx*, in Nile tilapia, further supporting the role of plant-derived additives in modulating antioxidant responses [34]. In the present study, the measured phenolic and flavonoid contents and DPPH radical-scavenging activity of WCE indicate that the crude extract contained antioxidant-active fractions. These properties may have contributed to the increased antioxidant enzyme activities and reduced lipid peroxidation observed in WCE-supplemented fish. Notably, the antioxidant responses were most evident at moderate inclusion levels, and the regression-derived response points were generally distributed within approximately 8–15 g kg^−1^ diet. This curvilinear pattern suggests that WCE may support antioxidant defense within a specific dietary range, whereas higher inclusion levels do not necessarily provide proportional additional benefits. However, targeted phytochemical profiling is still needed to identify the major compounds responsible for these effects.

Dietary WCE also improved innate immune-associated biomarkers at mucosal, humoral, and cellular levels, as indicated by increased mucus and serum lysozyme and peroxidase activities, higher ACH50, and improved phagocytic index and respiratory burst activity. Lysozyme and peroxidase are important antimicrobial enzymes in fish mucus and serum, whereas ACH50 reflects complement-mediated humoral activity. In parallel, higher phagocytic activity and respiratory burst indicate improved leukocyte responsiveness. Similar immunostimulatory effects have been reported in Nile tilapia fed extracts of *Camellia sinensis*, *Viscum album coloratum*, *Ocimum basilicum*, *Moringa oleifera*, *Psidium guajava*, *Tridax procumbens*, *Elephantopus scaber*, *Mitracarpus scaber*, *Avicennia marina*, and *Annona squamosa*, as well as *Azolla caroliniana* meal. These plant-derived additives improved one or more innate immune-associated parameters, including mucosal and serum lysozyme and peroxidase activities, complement activity, phagocytic responses, and respiratory burst activity [31,34,35,36,37,38,39,40,41]. In the present study, the favorable changes in these biomarkers may be partly related to the antioxidant-active fractions present in WCE, as reflected by its measured phenolic and flavonoid contents and DPPH radical-scavenging activity. By reducing oxidative pressure and maintaining cellular responsiveness, these antioxidant properties may help support basal immune-related functions. This interpretation is consistent with the curvilinear immune-associated responses observed in the regression analyses, where the estimated response points were mainly located within approximately 10–15 g kg^−1^ diet at week 4 and 10–14 g kg^−1^ diet at week 8. Therefore, the effects of WCE on basal innate immune-associated biomarkers appear to be most pronounced within a moderate inclusion range rather than increasing continuously with dose. However, because no pathogen challenge was conducted, the present findings should be interpreted as improved basal innate immune-associated responses rather than direct evidence of enhanced disease resistance.

The gene expression results provide additional molecular support for the antioxidant and innate immune-associated responses observed in this study. For immune-related genes, dietary WCE modulated the relative expression of *IL-1β*, *IL-8*, and *LBP* in the liver, suggesting possible effects on inflammatory regulation, leukocyte-related responses, and bacterial recognition under non-challenged conditions. In the intestine, WCE significantly affected *IL-8* and *LBP* expression, whereas *IL-1β* showed only a numerical increase and was not significantly different among treatments. This tissue-dependent response may reflect differences between the liver and intestine in basal immune regulation, with the liver responding more clearly to systemic metabolic and redox-related signals, while intestinal *IL-1β* may remain more tightly regulated under non-challenged conditions. Comparable modulation of immune- or cytokine-related genes has been reported in Nile tilapia supplemented with plant-derived additives such as *Ocimum basilicum*, *Avicennia marina*, *Juniperus communis*, *Moringa oleifera*, *Schinus terebinthifolius*, *Ginkgo biloba*, and *Phyllanthus emblica* [33,34,41,42,43,44,45]. These studies reported changes in genes such as *IL-1β*, *IL-8*, *TNF-α*, *IL-10*, *IFN-γ*, and *IgM*, although the direction of regulation differed among additives, tissues, doses, and experimental conditions.

For antioxidant-related genes, WCE affected *GSTα*, *GPX*, and *GSR*, which are involved in detoxification and glutathione-dependent redox regulation. These transcriptional responses were broadly consistent with the increased antioxidant enzyme activities and reduced MDA levels observed in serum and liver. Similar antioxidant-related gene responses have been reported in Nile tilapia fed *Avicennia marina* extract, *Juniperus communis* extract, and *Phyllanthus emblica* powder, where genes such as *SOD*, *CAT*, and *GPx*/*GSH-Px* were modulated alongside improved antioxidant status [34,41,42]. However, because qPCR results do not necessarily reflect protein abundance or enzyme activity, these findings should be interpreted as supportive molecular evidence rather than direct confirmation of pathway activation. Further studies combining phytochemical profiling, protein-level validation, and pathway-focused analyses are needed to clarify the mechanisms underlying WCE activity.

Overall, the present study indicates that WCE may serve as a plant-derived functional additive for Nile tilapia by improving growth performance, feed utilization, antioxidant status, basal innate immune-associated biomarkers, and related gene expression under controlled conditions. The most consistent responses observed in WCE10, together with the plateau or decline at WCE15 and WCE20, suggest a non-linear response typical of crude phytogenic extracts. At higher inclusion levels, changes in the balance among bioactive constituents may have contributed to the less consistent responses; however, the physiological mechanisms underlying this pattern were not investigated in the present study. Therefore, WCE appears to be more effective within a moderate inclusion range rather than showing a continuous dose-dependent improvement. Nevertheless, further studies are required to validate its practical application, including farm-scale trials, pathogen-challenge assays, digestive enzyme and nutrient digestibility analyses, gut microbiota profiling, intestinal and hepatic histology, protein-level immune markers, and standardized phytochemical characterization.

## 5. Conclusions

In summary, dietary WCE supplementation improved growth performance and feed utilization in Nile tilapia and was accompanied by favorable changes in antioxidant status, selected basal innate immune-associated biomarkers, and the relative expression of immune- and antioxidant-related genes. Among the tested diets, WCE10 produced the most consistent overall response, whereas higher inclusion levels showed less consistent benefits across the measured endpoints. Somatic indices were not significantly affected, indicating no marked alteration in gross visceral organ-weight allocation during the feeding trial. However, because digestive enzyme activity, nutrient digestibility, gut microbiota, intestinal morphology, and pathogen resistance were not directly assessed, the mechanisms underlying the observed growth-related responses and their relevance to disease outcomes remain to be clarified. Overall, WCE shows potential as a plant-derived feed additive for Nile tilapia diets, but further studies involving pathogen-challenge assays, mechanistic evaluation of digestive and intestinal responses, phytochemical standardization, diet stability assessment, and practical farming conditions are needed to confirm its efficacy and refine the optimal inclusion level.

## Figures and Tables

**Table 1:** Extraction yield and selected bioactive characteristics of *W. chinensis* extract used in the experiment.

Test Items	Results	Units	Analytical Basis	Methods
Crude extract yield	12.56 ± 2.85	%	Dry plant material	Ethanol maceration
Total phenolic content	3.70 ± 0.20	mg GAE g^−1^	Dry plant material	Folin–Ciocalteu method
Total flavonoid content	68.67 ± 2.84	mg QE g^−1^	Crude *W. chinensis* extract	Aluminum chloride colorimetric assay
Antioxidant activity	6.90 ± 0.50	mg vitamin E equivalents g^−1^	Dry plant material	DPPH assay

Note: Values are expressed as mean ± SD. GAE, gallic acid equivalents; QE, quercetin equivalents; DPPH, 2,2-diphenyl-1-picrylhydrazyl. Dry material refers to the dried *W. chinensis* material used for extraction, whereas crude extract refers to the concentrated ethanolic extract obtained after solvent removal.

**Table 2:** Formulation of the experimental diets.

Ingredients	WCE0	WCE5	WCE10	WCE15	WCE20
Fish meal	200	200	200	200	200
Soybean meal	390	390	390	390	390
Corn meal	150	150	150	150	150
Rice bran	150	150	150	150	150
Wheat flour	70	65	60	55	50
Soybean oil	5	5	5	5	5
WCE ^a^	0	5	10	15	20
Binder	5	5	5	5	5
Premix ^b^	25	25	25	25	25
Vitamin C 98%	5	5	5	5	5
Total	1000	1000	1000	1000	1000
**Proximate composition (% dry matter basis)**					
Dry matter	98.30	98.28	98.27	98.27	98.26
Gross energy (cal g^−1^)	4210.80	4210.62	4210.13	4209.90	4209.84
Crude protein	32.22	32.16	32.12	32.10	32.07
Ash	7.18	7.22	7.23	7.25	7.25
Fiber	3.90	3.91	3.91	3.92	3.93
Crude lipid	2.56	2.57	2.57	2.58	2.59

^a^ WCE: *Wedelia chinensis* extract. ^b^ Vitamin–mineral premix was added at 25 g kg^−1^ diet. The premix provided the following nutrients per kg premix: cholecalciferol, 217,000 IU; retinyl acetate, 1,085,000 IU; thiamine nitrate, 0.5 g; folic acid, 0.05 g; calcium pantothenate, 1.0 g; DL-α-tocopheryl acetate, 0.5 g; inositol, 0.5 g; pyridoxine hydrochloride, 0.5 g; niacin, 3.0 g; zinc, 1.0 g; copper, 0.25 g; manganese, 1.32 g; iodine, 0.05 g; cyanocobalamin, 10 mg; and sodium phosphate, 7.85 g.

**Table 3:** Specific primer sequences used for quantitative RT-PCR.

Target Gene	Sequence (5′–3′)	T_m_ (°C)	Product Size (bp)	Genbank No
*18S rRNA*	F: GTG*CAT*GGCCGTTCTTAGTT R: CTCAATCTCGTGTGGCTGAA	60	150	XR_003216134
*IL-1β*	F: GTCTGTCAAGGATAAGCGCTG R: ACTCTGGAGCTGGATGTTGA	59	200	XM_019365844
*IL-8*	F: CTGTGAAGG*CAT*GGGTGTG R: GATCACTTTCTTCACCCAGGG	59	196	NM_001279704
*LBP*	F: ACCAGAAACTGCGAGAAGGA R: GATTGGTGGTCGGAGGTTTG	59	200	XM_013271147
*GSTα*	F: ACTGCACACT*CAT*GGGAACA R: TTAAAAGCCAGCGGATTGAC	60	190	NM_001279635
*GPX*	F: GGTGGATGTGAATGGAAAGG R: CTTGTAAGGTTCCCCGTCAG	60	190	NM_001279711
*GSR*	F: CTGCACCAAAGAACTGCAAA R: CCAGAGAAGGCAGTCCACTC	60	172	XM_005467348

**Table 4:** Growth performance, feed utilization, and survival of Nile tilapia fed diets supplemented with different levels of WCE after 4 and 8 weeks of feeding.

	WCE0	WCE5	WCE10	WCE15	WCE20
**IW (g)**	14.33 ± 0.08	14.25 ± 0.05	14.33 ± 0.08	14.32 ± 0.08	14.33 ± 0.08
**FW (g)**					
4 weeks	31.12 ± 0.26 ^b^	32.70 ± 0.18 ^a^	33.12 ± 0.44 ^a^	32.83 ± 0.76 ^a^	32.65 ± 0.85 ^a^
8 weeks	64.23 ± 0.68 ^b^	69.23 ± 0.49 ^a^	70.15 ± 0.83 ^a^	69.03 ± 0.57 ^a^	68.83 ± 1.20 ^a^
**WGR (%)**					
4 weeks	117.10 ± 2.82 ^b^	129.47 ± 1.30 ^a^	131.06 ± 4.29 ^a^	129.33 ± 4.84 ^a^	127.80 ± 6.15 ^ab^
8 weeks	348.15 ± 5.37 ^b^	385.85 ± 2.30 ^a^	389.43 ± 6.93 ^a^	382.21 ± 6.25 ^a^	380.23 ± 7.47 ^a^
**SGR (%/day)**					
4 weeks	2.58 ± 0.04 ^b^	2.77 ± 0.02 ^a^	2.79 ± 0.06 ^a^	2.77 ± 0.07 ^a^	2.74 ± 0.09 ^ab^
8 weeks	2.50 ± 0.02 ^b^	2.63 ± 0.01 ^a^	2.65 ± 0.02 ^a^	2.62 ± 0.02 ^a^	2.62 ± 0.03 ^a^
**FCR**					
4 weeks	1.20 ± 0.03 ^a^	1.08 ± 0.01 ^b^	1.07 ± 0.03 ^b^	1.08 ± 0.04 ^b^	1.10 ± 0.05 ^b^
8 weeks	1.28 ± 0.02 ^a^	1.20 ± 0.01 ^b^	1.19 ± 0.02 ^b^	1.21 ± 0.03 ^b^	1.21 ± 0.01 ^b^
**SR (%)**					
4 weeks	100	100	100	100	100
8 weeks	96.67 ± 2.89	96.67 ± 5.77	95.00 ± 0.00	96.67 ± 2.89	96.67 ± 5.77

Note: Values are presented as mean ± SE (*n* = 3). Within a row, values with different superscript letters differ significantly (*p* < 0.05). WCE, *W. chinensis* extract; IW, initial weight; FW, final weight; WGR, weight gain rate; SGR, specific growth rate; FCR, feed conversion ratio; SR, survival rate.

**Table 5:** Somatic indices of Nile tilapia fed diets supplemented with different levels of WCE after 8 weeks of feeding.

Parameters	WCE0	WCE5	WCE10	WCE15	WCE20
HSI (%)	3.86 ± 0.16	3.96 ± 0.07	4.12 ± 0.01	3.83 ± 0.60	3.85 ± 0.14
VSI (%)	15.18 ± 0.44	15.95 ± 0.07	16.00 ± 0.27	15.58 ± 0.60	15.32 ± 0.43
ISI (%)	4.94 ± 0.08	4.97 ± 0.17	5.00 ± 0.18	4.98 ± 0.10	4.88 ± 0.14

Note: Values are presented as mean ± SE (*n* = 3). No significant differences were observed among treatments (*p* > 0.05). WCE, *W. chinensis* extract; HSI, hepatosomatic index; VSI, viscerosomatic index; ISI, intestinosomatic index.

**Table 6:** Serum and hepatic antioxidant enzyme activities and malondialdehyde levels of Nile tilapia fed diets supplemented with different levels of WCE after 8 weeks of feeding.

Items	Parameters	WCE0	WCE5	WCE10	WCE15	WCE20
Serum	*CAT* (U mL^−1^)	11.67 ± 0.37 ^b^	11.72 ± 0.26 ^ab^	12.19 ± 0.23 ^a^	11.41 ± 0.54 ^ab^	11.54 ± 0.45 ^ab^
	*SOD* (U mL^−1^)	14.72 ± 0.83 ^b^	15.93 ± 0.23 ^ab^	16.03 ± 0.16 ^a^	15.29 ± 0.90 ^ab^	15.14 ± 0.65 ^ab^
	MDA (nmol mL^−1^)	24.45 ± 0.88 ^a^	21.94 ± 1.59 ^ab^	20.61 ± 1.29 ^b^	21.14 ± 0.62 ^b^	21.92 ± 2.36 ^ab^
Liver	*CAT* (U mg^−1^ protein)	19.75 ± 0.82 ^b^	21.59 ± 1.84 ^ab^	23.06 ± 1.52 ^a^	20.89 ± 0.95 ^ab^	19.84 ± 1.86 ^b^
	*SOD* (U mg^−1^ protein)	14.48 ± 0.59 ^b^	15.45 ± 1.32 ^ab^	16.44 ± 0.59 ^a^	15.56 ± 0.67 ^ab^	15.23 ± 0.32 ^ab^
	MDA (nmol mg^−1^ protein)	20.49 ± 1.43 ^a^	19.12 ± 0.64 ^ab^	18.37 ± 0.17 ^b^	19.48 ± 0.59 ^ab^	19.81 ± 1.77 ^ab^
	*GPx* (U g^−1^ protein)	32.38 ± 1.45 ^c^	35.20 ± 2.68 ^bc^	38.88 ± 1.24 ^a^	38.36 ± 1.69 ^ab^	38.10 ± 1.39 ^ab^

Note: Values are presented as mean ± SE (*n* = 3). Within the same row, values with different superscript letters differ significantly (*p* < 0.05). WCE, *W. chinensis* extract; *CAT*, catalase; *SOD*, superoxide dismutase; MDA, malondialdehyde; *GPx*, glutathione peroxidase. Units are expressed as U mL^−1^ for serum *CAT* and *SOD*, nmol mL^−1^ for serum MDA, U mg^−1^ protein for hepatic *CAT* and *SOD*, nmol mg^−1^ protein for hepatic MDA, and U g^−1^ protein for hepatic *GPx*.

**Table 7:** Innate immune responses of Nile tilapia fed diets supplemented with different levels of WCE after 4 and 8 weeks of feeding.

Parameter	WCE0	WCE5	WCE10	WCE15	WCE20
4 weeks					
SMLA	1.21 ± 0.04 ^b^	1.42 ± 0.09 ^a^	1.41 ± 0.10 ^a^	1.40 ± 0.05 ^ab^	1.33 ± 0.08 ^ab^
SMPA	0.15 ± 0.03 ^b^	0.19 ± 0.03 ^ab^	0.23 ± 0.02 ^a^	0.19 ± 0.06 ^ab^	0.16 ± 0.05 ^ab^
SLA	2.68 ± 0.17 ^b^	2.91 ± 0.16 ^ab^	3.09 ± 0.19 ^a^	3.01 ± 0.15 ^a^	2.79 ± 0.14 ^ab^
SPA	0.10 ± 0.02 ^b^	0.13 ± 0.03 ^ab^	0.17 ± 0.01 ^a^	0.12 ± 0.05 ^ab^	0.13 ± 0.03 ^ab^
ACH50	112.76 ± 2.55 ^c^	115.13 ± 3.03 ^bc^	120.90 ± 2.89 ^a^	120.42 ± 1.78 ^a^	119.45 ± 1.87 ^ab^
PI	1.64 ± 0.08	1.68 ± 0.13	1.80 ± 0.06	1.75 ± 0.06	1.77 ± 0.13
RB	0.10 ± 0.02 ^c^	0.15 ± 0.03 ^abc^	0.19 ± 0.01 ^b^	0.17 ± 0.02 ^ab^	0.13 ± 0.06 ^bc^
8 weeks					
SMLA	1.55 ± 0.07 ^b^	1.86 ± 0.17 ^ab^	2.01 ± 0.14 ^a^	1.97 ± 0.11 ^a^	1.88 ± 0.19 ^ab^
SMPA	0.14 ± 0.02 ^b^	0.17 ± 0.02 ^ab^	0.21 ± 0.02 ^a^	0.19 ± 0.04 ^a^	0.18 ± 0.02 ^ab^
SLA	4.05 ± 0.19 ^b^	4.16 ± 0.25 ^b^	5.02 ± 0.04 ^a^	4.56 ± 0.68 ^ab^	4.27 ± 0.72 ^ab^
SPA	0.17 ± 0.01 ^b^	0.22 ± 0.04 ^ab^	0.26 ± 0.03 ^a^	0.20 ± 0.08 ^ab^	0.21 ± 0.03 ^ab^
ACH50	124.87 ± 4.13 ^b^	132.71 ± 4.82 ^ab^	136.17 ± 1.40 ^a^	131.33 ± 7.91 ^ab^	131.55 ± 5.92 ^ab^
PI	2.19 ± 0.06 ^b^	2.39 ± 0.31 ^ab^	2.74 ± 0.10 ^a^	2.59 ± 0.05 ^ab^	2.33 ± 0.37 ^b^
RB	0.19 ± 0.05 ^c^	0.22 ± 0.02 ^bc^	0.32 ± 0.04 ^a^	0.31 ± 0.05 ^a^	0.28 ± 0.03 ^ab^

Note: Values are presented as mean ± SE (*n* = 3). Within the same row, values with different superscript letters differ significantly (*p* < 0.05). WCE, *W. chinensis* extract; SMLA, skin mucus lysozyme activity; SMPA, skin mucus peroxidase activity; SLA, serum lysozyme activity; SPA, serum peroxidase activity; ACH50, alternative complement pathway activity; PI, phagocytic index; RB, respiratory burst activity. Units are expressed as μg mL^−1^ for SMLA and SLA, U mL^−1^ for SMPA and SPA, U mL^−1^ for ACH50, and OD_655_ for RB.

## Data Availability

The data supporting the findings of this study are available from the corresponding author upon reasonable request.

## Supplementary Materials

The following supporting information can be downloaded at: https://www.mdpi.com/article/10.3390/ani16131986/s1, Table S1: Primer amplification efficiency and melt-curve specificity for the qRT-PCR assays. Table S2: RNA quality characteristics and stability assessment of the *18S rRNA* reference gene. Figure S1: Polynomial regression plots for antioxidant parameters in Nile tilapia fed diets supplemented with different levels of WCE. (A) Serum *CAT*; (B) serum *SOD*; (C) serum *MDA*; (D) liver *CAT*; (E) liver *SOD*; (F) liver MDA; (G) liver *GPx*. WCE, *W*. chinensis extract; *CAT*, catalase; *SOD*, superoxide dismutase; MDA, malondialdehyde; *GPx*, glutathione peroxidase. Figure S2: Polynomial regression plots for innate immune-associated parameters in Nile tilapia fed diets supplemented with different levels of WCE at week 4. (A) Skin mucus lysozyme activity; (B) skin mucus peroxidase activity; (C) serum lysozyme activity; (D) serum peroxidase activity; (E) alternative complement activity; (F) phagocytic index; (G) respiratory burst activity. WCE, W. chinensis extract; SMLA, skin mucus lysozyme activity; SMPA, skin mucus peroxidase activity; SLA, serum lysozyme activity; SPA, serum peroxidase activity; ACH50, alternative complement activity; PI, phagocytic index; RB, respiratory burst activity. Figure S3: Polynomial regression plots for innate immune-associated parameters in Nile tilapia fed diets supplemented with different levels of WCE at week 8. (A) Skin mucus lysozyme activity; (B) skin mucus peroxidase activity; (C) serum lysozyme activity; (D) serum peroxidase activity; (E) alternative complement activity; (F) phagocytic index; (G) respiratory burst activity. WCE, W. chinensis extract; SMLA, skin mucus lysozyme activity; SMPA, skin mucus peroxidase activity; SLA, serum lysozyme activity; SPA, serum peroxidase activity; ACH50, alternative complement activity; PI, phagocytic index; RB, respiratory burst activity. Figure S4: Derivative melt-curve profiles of qRT-PCR amplicons generated with primers for (A) *18S rRNA*, (B) *IL-1β*, (C) *IL-8*, (D) *LBP*, (E) *GSTα*, (F) *GPX*, and (G) *GSR*.
