# Supplementary material for: Effects of Dietary Wedelia chinensis (Osbeck) Merr. Extract on Growth Performance, Feed Utilization, Antioxidant Status, and Innate Immunity in Nile Tilapia (Oreochromis niloticus)
Source: Animals (Basel). 2026 Jun 27;16(13):1986. doi: 10.3390/ani16131986 (PMC13359914; doi:10.3390/ani16131986)
Supplement: Supplementary file 1 [file animals-16-01986-s001.zip › animals-4358165-supplementary.pdf]

Supplementary Materials

Effects of Dietary *Wedelia chinensis* (Osbeck) Merr. Extract on Growth Performance, Feed Utilization, Antioxidant Status, and Innate Immunity in Nile Tilapia (*Oreochromis niloticus*)

Table S1. Primer amplification efficiency and melt-curve specificity for the qRT-PCR assays.

No	Gene	Slope	R²	Amplification efficiency (%)	Melt-curve specificity
1	*18S rRNA*	−3.352	0.9998	98.8	Predominant gene-specific peak observed
2	*IL-1β*	−3.299	0.9999	101.0	Predominant gene-specific peak observed
3	*IL-8*	−3.361	0.9996	98.4	Predominant gene-specific peak observed
4	*LBP*	−3.443	0.9999	95.2	Predominant gene-specific peak observed
5	*GSTα*	−3.234	0.9997	103.8	Predominant gene-specific peak observed
6	*GPX*	−3.398	1.0000	96.9	Predominant gene-specific peak observed
7	*GSR*	−3.339	0.9999	99.3	Predominant gene-specific peak observed

Note: Primer efficiency was calculated from five-point cDNA dilution curves. Melting-curve analysis was evaluated for each primer pair.

Table S2. RNA quality characteristics and stability assessment of the 18S rRNA reference gene.

A. RNA quality of samples used for cDNA synthesis

No	Tissue	n	RNA concentration (ng μL⁻¹)	A260/A280	A260/A230
1	Liver	45	654.6–1133.3	1.92–2.07	1.79–2.20
2	Intestine	45	355.5–641.9	1.88–2.05	1.81–2.15

B. 18S rRNA Ct stability across dietary treatments

No	Tissue	WCE0	WCE5	WCE10	WCE15	WCE20	F	P
1	Liver	13.661 ± 0.177	13.564 ± 0.152	13.680 ± 0.107	13.574 ± 0.344	13.621 ± 0.085	0.207	0.929
2	Intestine	14.394 ± 0.196	14.302 ± 0.137	14.254 ± 0.171	14.423 ± 0.160	14.438 ± 0.154	0.707	0.605

Note: RNA quality values are expressed as the observed range across samples used for cDNA synthesis (n = 45 per tissue). Ct values are expressed as mean ± SD of tank means (n = 3 tanks per dietary treatment). One-way ANOVA was performed separately for liver and intestine.

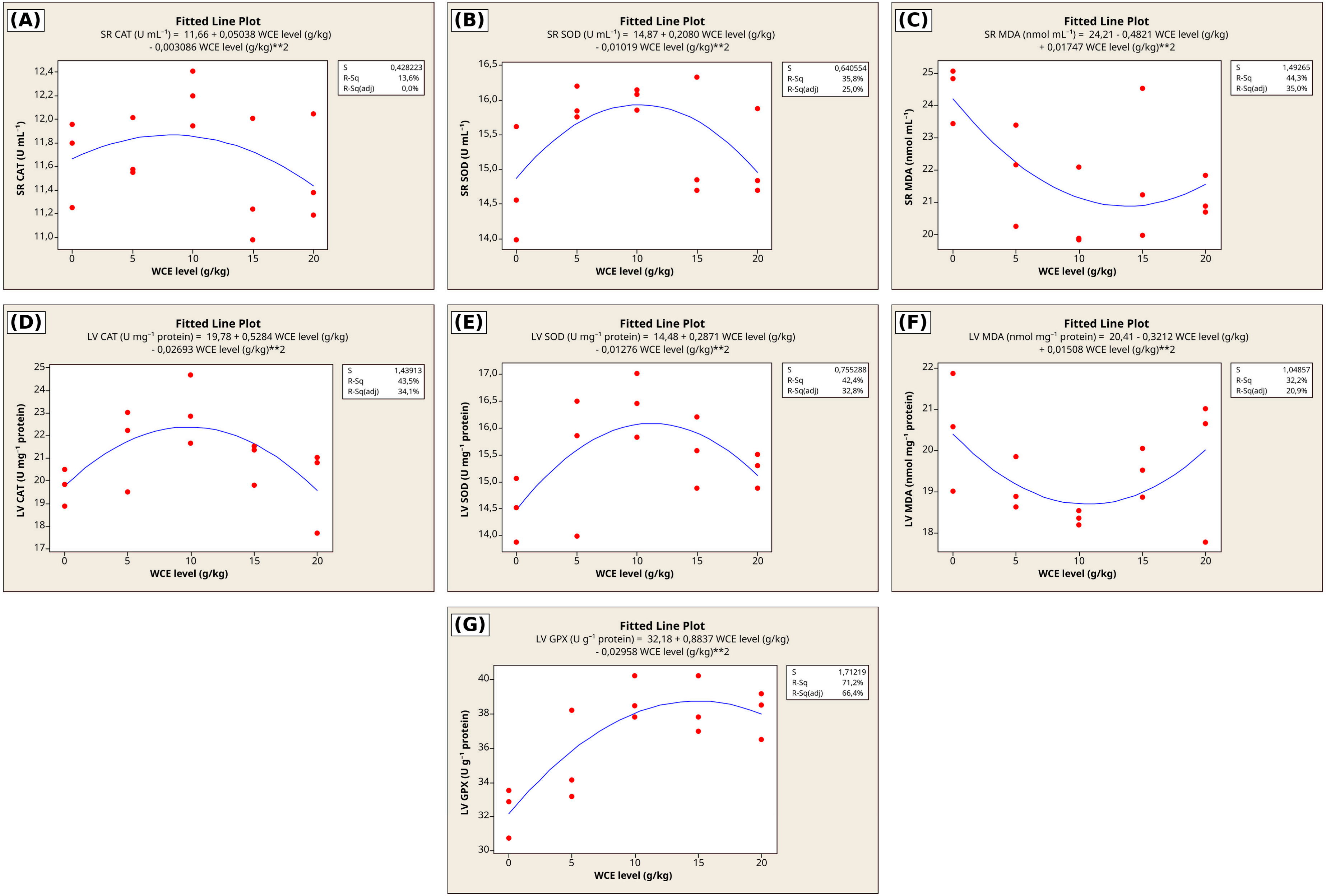

Supplementary Figure S1. Polynomial regression plots for antioxidant parameters in Nile tilapia fed diets supplemented with different levels of WCE. (A) Serum *CAT*; (B) serum *SOD*; (C) serum *MDA*; (D) liver *CAT;* (E) liver *SOD*; (F) liver MDA; (G) liver *GPx*. WCE, *W. chinensis* extract; *CAT*, catalase; *SOD*, superoxide dismutase; MDA, malondialdehyde; *GPx*, glutathione peroxidase.

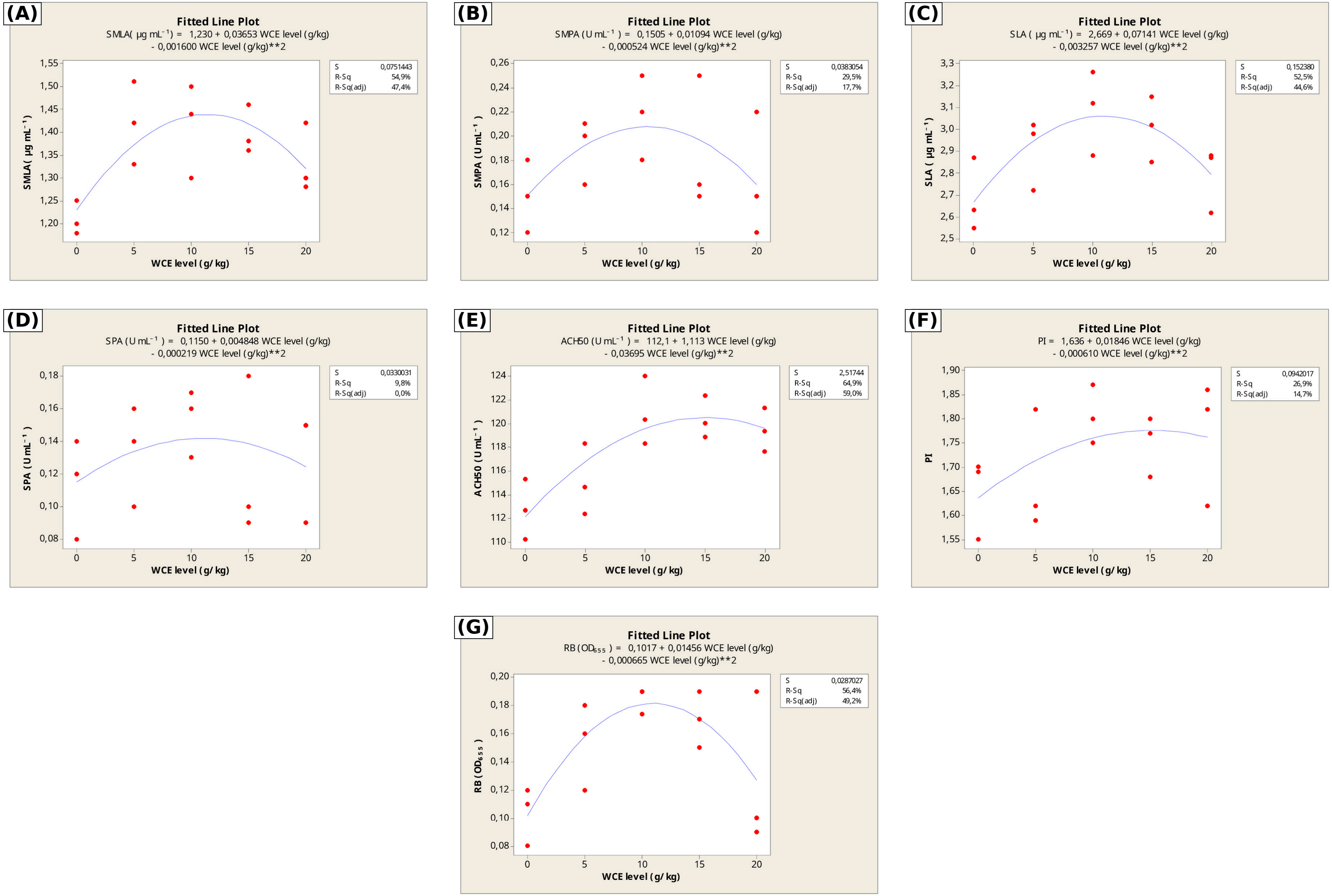

Supplementary Figure S2. Polynomial regression plots for innate immune-associated parameters in Nile tilapia fed diets supplemented with different levels of WCE at week 4. (A) Skin mucus lysozyme activity; (B) skin mucus peroxidase activity; (C) serum lysozyme activity; (D) serum peroxidase activity; (E) alternative complement activity; (F) phagocytic index; (G) respiratory burst activity. WCE, W. chinensis extract; SMLA, skin mucus lysozyme activity; SMPA, skin mucus peroxidase activity; SLA, serum lysozyme activity; SPA, serum peroxidase activity; ACH50, alternative complement activity; PI, phagocytic index; RB, respiratory burst activity.

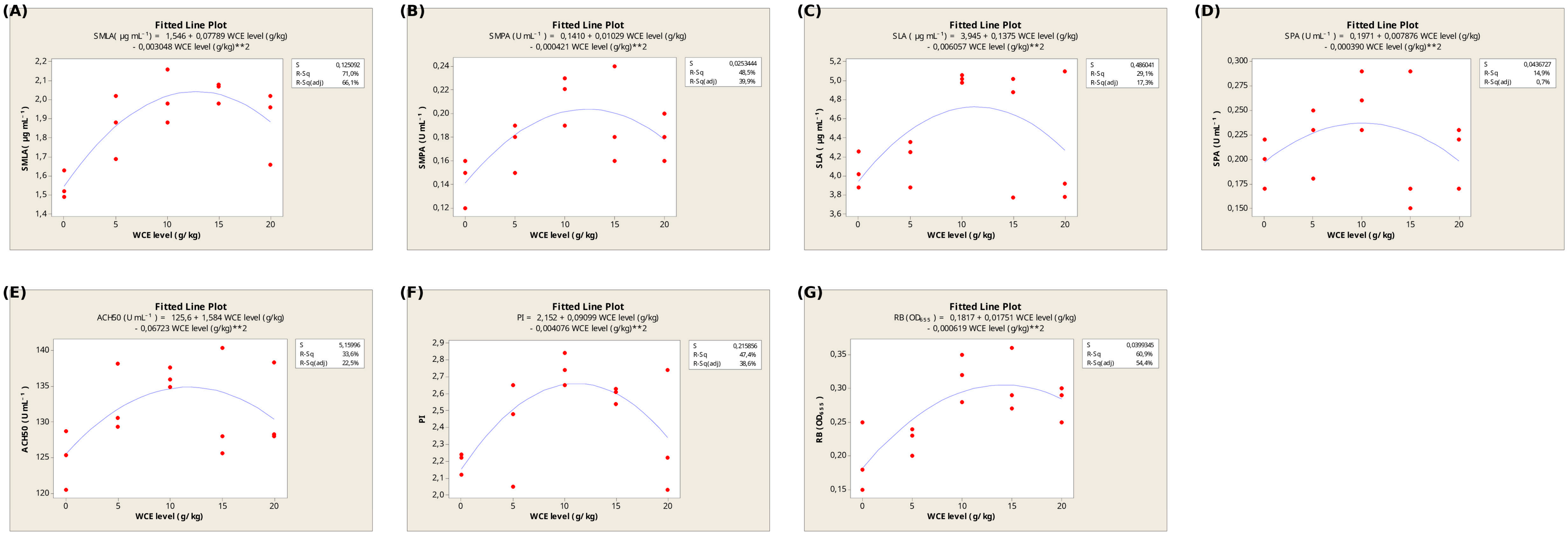

Supplementary Figure S3. Polynomial regression plots for innate immune-associated parameters in Nile tilapia fed diets supplemented with different levels of WCE at week 8. (A) Skin mucus lysozyme activity; (B) skin mucus peroxidase activity; (C) serum lysozyme activity; (D) serum peroxidase activity; (E) alternative complement activity; (F) phagocytic index; (G) respiratory burst activity. WCE, W. chinensis extract; SMLA, skin mucus lysozyme activity; SMPA, skin mucus peroxidase activity; SLA, serum lysozyme activity; SPA, serum peroxidase activity; ACH50, alternative complement activity; PI, phagocytic index; RB, respiratory burst activity.

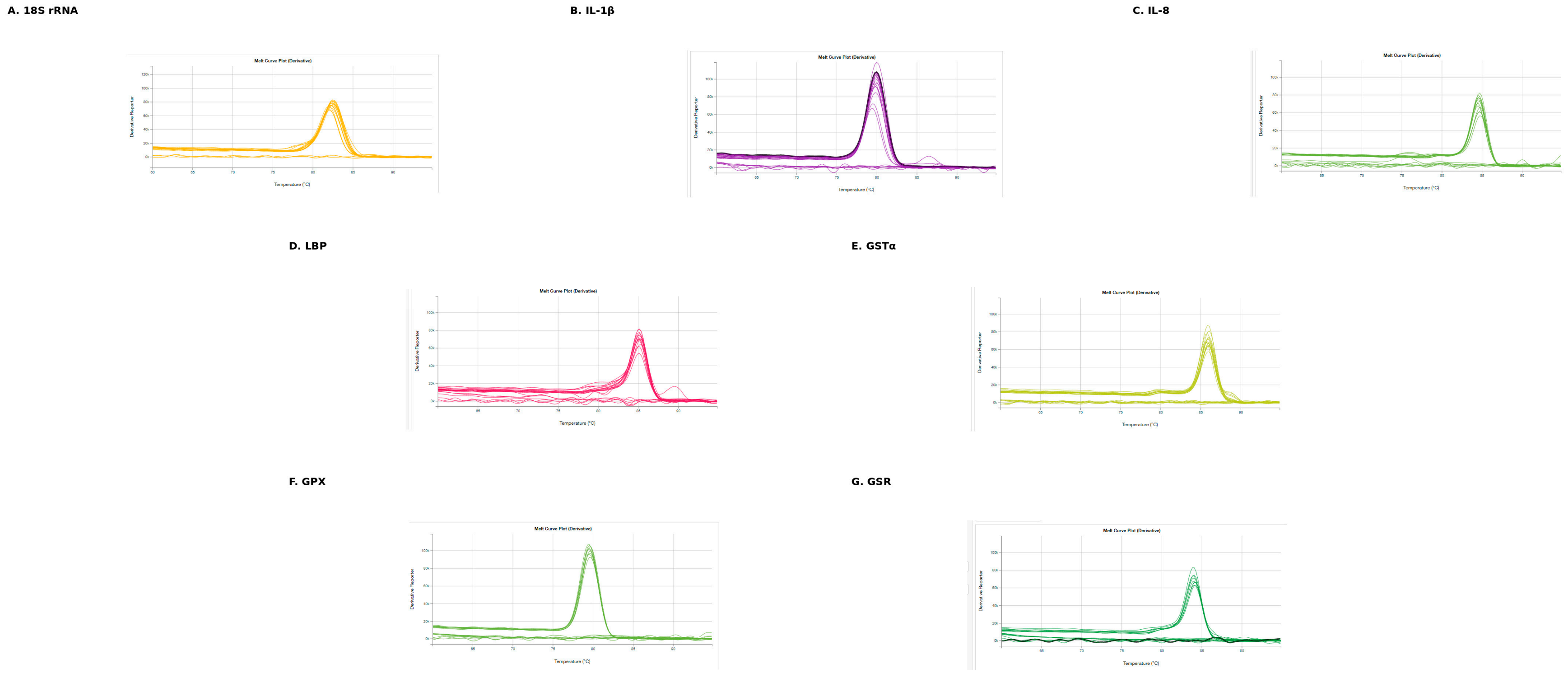

Supplementary Figure S4. Derivative melt-curve profiles of qRT-PCR amplicons generated with primers for (A) *18S rRNA*, (B) *IL-1β*, (C) *IL-8*, (D) *LBP*, (E) *GSTα*, (F) *GPX*, and (G) *GSR*.

A predominant gene-specific melt peak was observed for each primer pair, supporting amplification specificity.
